# Uniparental disomy as a mechanism for *CERS3*‐mutated autosomal recessive congenital ichthyosis

**DOI:** 10.1111/bjd.16999

**Published:** 2018-09-20

**Authors:** S. Polubothu, M. Glover, S.E. Holder, V.A. Kinsler

**Affiliations:** ^1^ Genetics and Genomic Medicine UCL Great Ormond Street Institute of Child Health London WC1N 1EH U.K; ^2^ Paediatric Dermatology Great Ormond Street Hospital for Children London WC1N 3JH U.K; ^3^ North West Thames Regional Genetics Service Kennedy Galton Centre London HA1 3UJ U.K

dear editor, An 8‐year‐old girl was under the care of dermatology with congenital ichthyosis. Her past history was of full‐term birth as a collodion baby, the first child of unrelated healthy parents. In the neonatal period she developed generalized ichthyosiform erythroderma. Poor feeding, hypotonia and mild dysmorphic features led to a clinical diagnosis of Prader–Willi syndrome (PWS) in infancy. Methylation‐specific polymerase chain reaction analysis at the *SNRPN* locus on chromosome 15q revealed absence of the paternal allele, confirming this diagnosis. Possible causes are a deletion of the paternal allele or maternal uniparental disomy (UPD);^1^, ^2^ however, microsatellite analysis confirmed that this was secondary to UPD, specifically maternal isodisomy of chromosome 15. Subsequently, and compatible with PWS, she had growth failure requiring growth hormone therapy, mild developmental delay and scoliosis.

**Figure 1:**
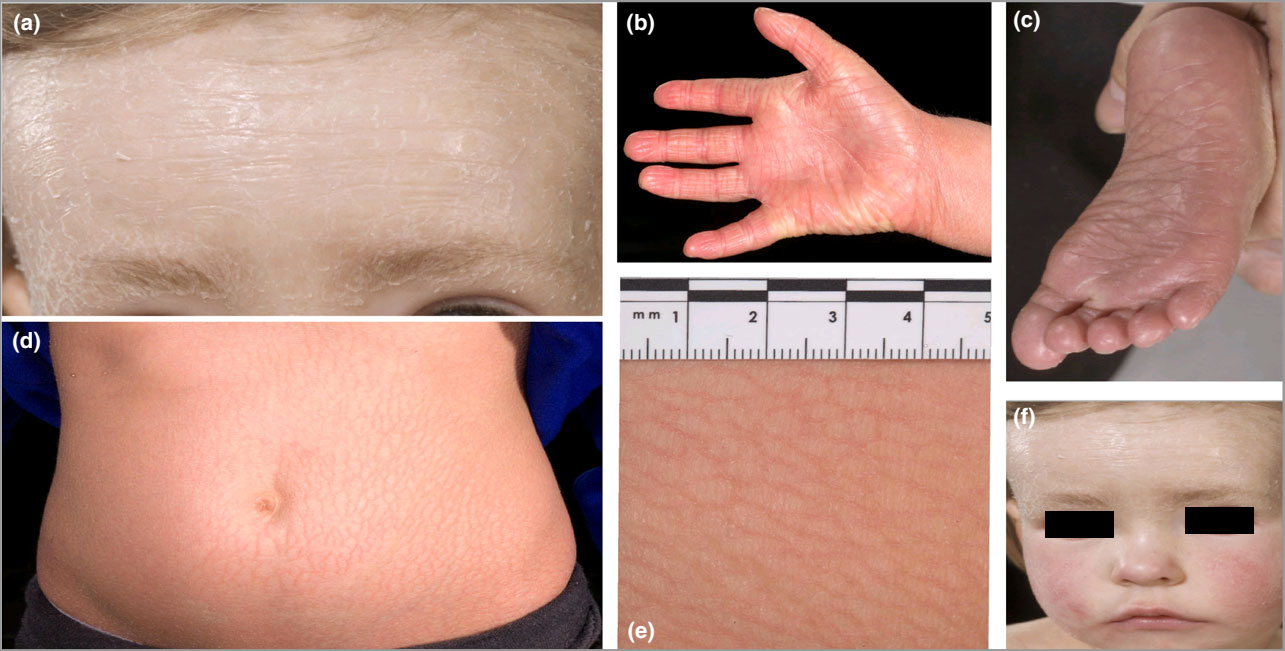
Clinical features. (a) Generalized fine scale of the face and (d, e) ichthyosiform erythroderma of the trunk. Additional features observed include hyperlinearity of the palms (b) and soles (c) and multiple acquired melanocytic naevi (f). The facial features were consistent with the characteristic facial features observed in Prader–Willi syndrome, including a narrow temple distance, almond‐shaped eyes and a thin upper lip with downturned mouth, as seen in (f).

On examination she had generalized ichthyosis with a fine scale and mild erythroderma. She additionally had palmoplantar hyperlinearity and 10–15 acquired melanocytic naevi (Fig. 1). Her height was on the 2nd centile and her weight on the 9–25th. Her history and clinical presentation were considered compatible with an autosomal recessive congenital ichthyosis (ARCI).

We hypothesized that her skin disease could be secondary to the known uniparental disomy, which pinpointed *CERS3* as the only known genecandidate on chromosome 15. Lymphocyte DNA was extracted and sequenced on a diagnostic‐grade targeted next‐generation sequencing panel for genes known to be mutated in Mendelian disorders of cornification (data available on request). This revealed a novel homozygous *CERS3* mutation, NM_001290342·2, c.716A≥T, p.(Asp239Val); NM_001290341·2, c.749A>T, p.(Asp250Val); and no other pathogenic variants. This mutation is predicted to be damaging *in silico*, with Polyphen2 and SIFT scores of 1 and 0·02, respectively. It also has a very low allele frequency in public databases (Exac 8·2 × 10^−6^, gnomAD 1·2 × 10^−5^). Analysis of lymphocyte DNA from both parents revealed the same variant in the patient's mother, as a heterozygous variant, further supporting maternal UPD(15) specifically with isodisomy as the underlying mechanism.

While the majority of genes in humans are biallelically expressed, genomic imprinting of at least 90 genes leads to monoallelic expression from one parental allele in healthy individuals. These imprinted genes tend to occur in clusters at genomic loci. UPD occurs when both copies of a chromosome or part of a chromosome are derived from the same parent. The most common mechanism leading to UPD in PWS is trisomic rescue, in which two maternal chromosomes are present in the egg fertilized by a normal haploid sperm. Trisomy 15 is not compatible with life, although a trisomy 15 rescue event can occur in which mitotic loss of one copy of chromosome 15 enables survival. The initial disomic event leading to maternal UPD can occur as a result of nondisjunction of chromosomes at different stages of meiosis, termed heterodisomy when each of the mother's two chromosomes are inherited, or isodisomy when two identical maternal chromosomes are inherited from the mother.

PWS was the first disorder reported to be caused by UPD, and is due to absence of expression of paternal genes in the imprinted region on the long arm of chromosome 15, 15q11·2‐q13.^3^

UPD has long been recognized as a genetic mechanism causing diseases that are classically autosomal recessive, first described in 1988 in a patient presenting with cystic fibrosis and maternal UPD of chromosome 7.^4^ This phenomenon has been described previously as causing dermatological disease, including Netherton syndrome, harlequin ichthyosis and epidermal bullosa. Given the proximity of *CERS3* to the PWS region it is highly likely that the phenotype of ARCI observed in our patient is attributable to maternal uniparental disomy of the mutated *CERS3*. In support of this hypothesis is the homozygosity for a novel mutation in a child of unrelated parents, the absence of other pathogenic mutations in ARCI genes, and the similarity of the phenotype to those of previous cases of *CERS3‐*mutated ARCI.

ARCI is a subset of nonsyndromic ichthyosis.^5^ There is considerable phenotypic variation between the different subtypes, and 11 causative genes have been described thus far. Wu and Lee first demonstrated an ARCI locus on chromosome 15q26·3, using whole‐genome homozygosity mapping in six patients from an aboriginal population in Southern Taiwan.^6^ Affected individuals had a collodion membrane at birth with progressive appearance of generalized fine erythrodermic scales and palmoplantar hyperlinearity. In 2013 Eckl *et al*. identified mutations in the gene *CERS3* located in this locus in a large consanguineous family (exon 4, c.43T>C, p.Trp15Arg).^7^ *CERS3* encodes ceramide synthase 3, a vital skin barrier protein. The original proband presented with moderate lamellar ichthyosis and mild erythroderma similar to our patient.

Additional features described include hypohidrosis and recurrent superficial skin infections. Four further patients with *CERS3*‐mutated ARCI were found to have homozygous deletions of exon 13, and a fifth had a homozygous exon 9 splice‐site mutation. This fifth patient was from a large consanguineous family presenting with homozygous contiguous gene deletion syndrome on chromosome 15q26·3.^8^ All five presented as collodion babies, and had generalized fine scale, erythema, hyperlinearity of the palms and premature ageing of the skin on the backs of the hands. Additionally, as observed in our patient, multiple acquired melanocytic naevi were seen in three of these patients and hyperlinearity of the soles in one.

In summary, we present a case of uniparental isodisomy on chromosome 15 resulting in both PWS and *CERS3*‐related ARCI. Clinicians should consider uniparental disomy as a mechanism for autosomal recessive diseases such as ARCI, particularly when presented with syndromes associated with UPD or in the absence of a history of consanguinity.
